# Acteoside: a novel green inhibitor for the corrosion of copper in 1.0 M HNO_3_ solution: experimental and theoretical investigation†

**DOI:** 10.1039/d5ra01657f

**Published:** 2025-03-27

**Authors:** Mahmoud A. Al-Qudah, Tareq T. Bataineh, Faten M. Abu Orabi, Sultan T. Abu-Orabi, Ghassab M. Al-Mazaideh, Abbas I. Alakhras

**Affiliations:** a Department of Chemistry, Faculty of Science, Yarmouk University P.O. Box 566 Irbid 21163 Jordan mahmoud.qudah@yu.edu.jo +96227211117 +96277420029; b Faculty of Arts and Sciences, The World Islamic Sciences and Education University Amman Jordan faten.aladwan@wise.edu.jo; c Department of Medical Analysis, Faculty of Science, Tishk International University Erbil KRG Iraq; d Department of Pharmaceutical Chemistry, College of Pharmacy, University of Hafr Al Batin P. O. Box: 1803 Hafr Al Batin 31991 Saudi Arabia gmazaideh@uhb.edu.sa; e Department of Chemistry, College of Science, Imam Mohammad Ibn Saud Islamic University (IMSIU) Riyadh 11623 Saudi Arabia

## Abstract

Acteoside (ACT) isolated from *A. orientalis* L. was investigated as a corrosion inhibitor of copper in 1.0 M HNO_3_ acid solutions using conventional weight loss, electrochemical polarization, and electrochemical impedance spectroscopy (EIS) studies. The molecular structure of acteoside (ACT) is supported by all the experimental results from LC-MS, FT-IR, ^1^H, and ^13^C-NMR. The findings indicate that ACT is a potent inhibitor, and that its effectiveness increases with both temperature and inhibitor concentration. The highest inhibitor concentration occurs at 48 °C, and the inhibition efficiency peaks at 98.8%. The results indicated that the presence of the inhibitor slightly lessened copper dissolution with rising temperature. ACT adheres to the Langmuir, Freundlich, El-Awady, Temkim, and Redlich–Peterson (R–P) adsorption isotherms on copper surfaces with a high regression coefficient value. The values of the activation parameters (*E*_a_, ΔH*, ΔS*) and the adsorption thermodynamic functions (Δ*G*_ads_) suggest both physisorption and chemisorption processes. Electrochemical polarization data were used to identify the mixed mode of inhibition. Active molecules adhere to the metal surface and form a protective layer, which causes changes in impedance characteristics, charge transfer resistance, and double layer capacitance in response to variations in ACT concentration. Weight loss and electrochemical data are supported by quantum chemical computations.

## Abstract 2

Acteoside (ACT) isolated from *A. orientalis* L. was investigated as a corrosion inhibitor of copper in 1.0 M HNO_3_ acid solutions using conventional weight loss, electrochemical polarization, and electrochemical impedance spectroscopy (EIS) studies.

## Graphical abstract

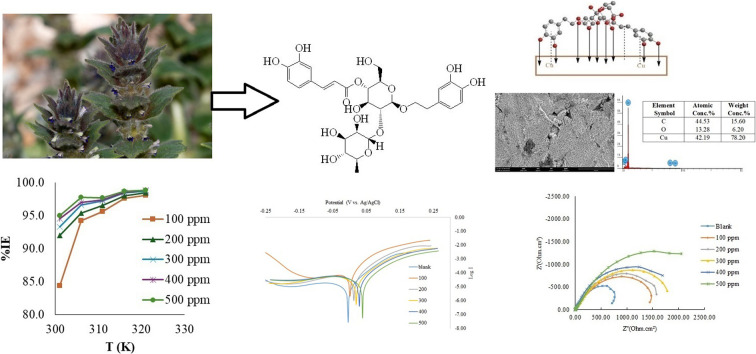

## 1. Introduction

Many corrosion problems can occur to copper during processing in acidic media. A practical and affordable method to prevent copper from corroding is to add corrosion inhibitors to acid solutions.^1,2^ Because of their low cost and strong corrosion resistance, organic corrosion inhibitors are frequently used. On the other side, several commonly used organic corrosion inhibitors have challenging synthesis processes, high toxicity, and are prone to environmental issues.

Natural products serve as copper corrosion inhibitors due to their environmental friendliness, affordability, and the presence of a variety of organic compounds, some of which have demonstrated their effectiveness as metal corrosion inhibitors by containing heteroatoms like O, N, S, and P.^3–10^ The use of extracts and isolated compounds from natural sources as corrosion inhibitors for metals is interesting. Previous studies have used natural extracts and compounds as corrosion inhibitors and demonstrated high effectiveness in preventing metal corrosion. Table 1 shows a comparison of the compound used in the study with other inhibitors, including organic inhibitors, plant extracts, and isolated natural compounds.^11–20^

**Table 1:** The inhibition efficiency of organic and natural inhibitors in a HNO_3_ solution on copper^21–31^

Inhibitors	[Inhibitors]	IE (%)	Note	Reference
Acteoside	100–500 ppm	84.3–98.8	The findings indicate that ACT is a potent inhibitor, and that its effectiveness increases with both temperature and inhibitor concentration	
3-Oxocostusic acid	4.0 × 10^−4^–16.0 × 10^−4^ mol L^−1^	Up to 95.60	The inhibition efficiency of 3-oxocostusic acid is high, it means that it effectively prevents copper from corroding in nitric acid	21
Alkaloids (quinine, nicotine)	10^−3^ M	50–85	These are nitrogen-containing heterocycles that form strong interactions with metal surfaces, providing inhibition	22
*Adhatoda vasica* leaf extract	0.01 to 0.1 g L^−1^	73–78	Spontaneous, governed by physiochemical processes, and occurred according to the Langmuir's adsorption isotherm	23
*Vitex negundo* leaf extract	0.01 to 0.1 g L^−1^	93–98	Spontaneous, governed by physiochemical processes, and occurred according to the Langmuir's adsorption isotherm	23
*Saraca asoca* leaf extract	0.01 to 0.1 g L^−1^	53–91	Spontaneous, governed by physiochemical processes, and occurred according to the Langmuir's adsorption isotherm	23
*Thymus satureioides* essential oil	1200–1600 ppm	69.72–89.04	The efficiency increases with the inhibitor concentration	24
*Rosmarinus officinalis* extract	300 ppm	77.0	The findings showed that when temperature rises, physisorption increases and inhibitory efficiency (%IE) decreases. The adsorption mechanism and Langmuir's adsorption model agreed	25
Amino acid (proline (Pro), phenylalanine (Phe), tyrosine (Tyr), and tryptophan (Try))	10^−3^ M	35–90	These amino acids can act as corrosion inhibitors by forming protective layers on the surface of copper, thereby reducing the corrosion rate when exposed to aggressive environments like nitric acid	26
Amino acids (histidine, glycine)	10^−3^–10^−2^ M	55–80	Slightly less efficient (55–80%) but have the advantage of being eco-friendly and biodegradable	27
Benzotriazole (BTA)	10^−3^–10^−2^ M	80–95	The highest inhibition efficiency (80–95%) due to its strong adsorption and formation of a protective film on the copper surface	28
2-Mercaptobenzothiazole (MBT)	10^−3^ M	70–90	Strong adsorption on Cu surface	29
Schiff bases	10^−3^–10^−2^ M	60–85	Moderately effective (60–85%) due to their chelation properties, though their efficiency depends on their molecular structure	30
Imidazole derivatives	10^−3^–10^−2^ M	75–92	Exhibit a similar range (75–92%) as BTA, benefiting from π-electron interactions that improve adsorption	31

To increase the efficacy of possible inhibitors, researchers have recently employed quantum chemistry techniques in corrosion inhibitor experiments.^32–34^ In this area, the combination of computer science and density functional theory (DFT) has produced useful instruments for examining the molecules of natural products. When compared to conventional post-Hartree–Fock (HF) approaches, the DFT methodology provides a better treatment of electron interactions. DFT has been widely utilized by researchers to quantify several aspects of substances, including their kinetics, thermochemistry, and chemical structure. These methods advance our knowledge of corrosion inhibition and could lead to the creation of future inhibitors that are more effective and long-lasting.

The isolation of natural compounds from plant extracts and analysis of their anticorrosion activity for metals is a new trend. The current study used weight loss, electrochemical polarization, EIS, and quantum chemical calculations techniques to investigate the inhibition of copper corrosion by ACT in a 1.0 M HNO_3_ solution.

## 2. Experimental

### 2.1. Materials and instrumentation

The purity of the copper sheet utilized, which was supplied by Goodfellow USA, was higher than 99.99%. The only foils exposed to the corrosive environment were the rectangular foils, which are the working electrode in a conventional three-electrode electrochemical cell. The samples of the copper sheet, measuring 3.0 cm by 1.0 cm by 0.50 mm, were cut out. All the chemicals used were AR-grade and came from various suppliers. Reagents were dissolved in triple-distilled water to create all solutions. CorrTest potentiostats (CS350, China) were used for electrochemical testing inside the corrosion cell. The counter electrode was a Pt electrode rod, and the reference electrode was silver/silver chloride. A Julabo SW22 thermostat was used to adjust the test's temperature.

### 2.2. Plant material

During the April 2022 flowering season, plants of *A. orientalis* L. were collected from the Ajloun area. Yarmouk University's Department of Biology, Faculty of Science, Irbid, Jordan's Prof. Dr Jamil N. Lahham verified the plant material's identity. The plant material (3.0 kg) was dried and ground into a fine powder. The powder was soaked in petroleum ether (40–60 °C, 30 Liters) at room temperature for 10 days to defatted it. The remaining defatted plant powder was then extracted using ethanol five times, each lasting seven days and thirty liters. About 125 g of crude residue was obtained by vacuum-evaporating. Water and chloroform were used to separate the crude ethanolic residue. After vacuum-evaporating the chloroform, the dry residue was separated into 10% aqueous methanol and hexane. Water was treated with *n*-butanol to remove polar organic molecules.

The aqueous methanol extract (75 g) was adsorbed on 75 g of silica gel S and subjected to column chromatography (40.0 × 6.0 cm) using 500 g of the same absorbent. Hexane was used to fill the column and to elute the sample. Then, ethyl acetate was added, gradually raising the polarity. According to their TLC behavior, a total of 60 fractions obtained using this method (each 500 mL) were divided into 4 major groups (AMPM I–AMPM IV). Each of these groups underwent CC and TLC purification or treatment with an appropriate solvent.

### 2.3. Acteoside (ACT)

**Fig. 1:**
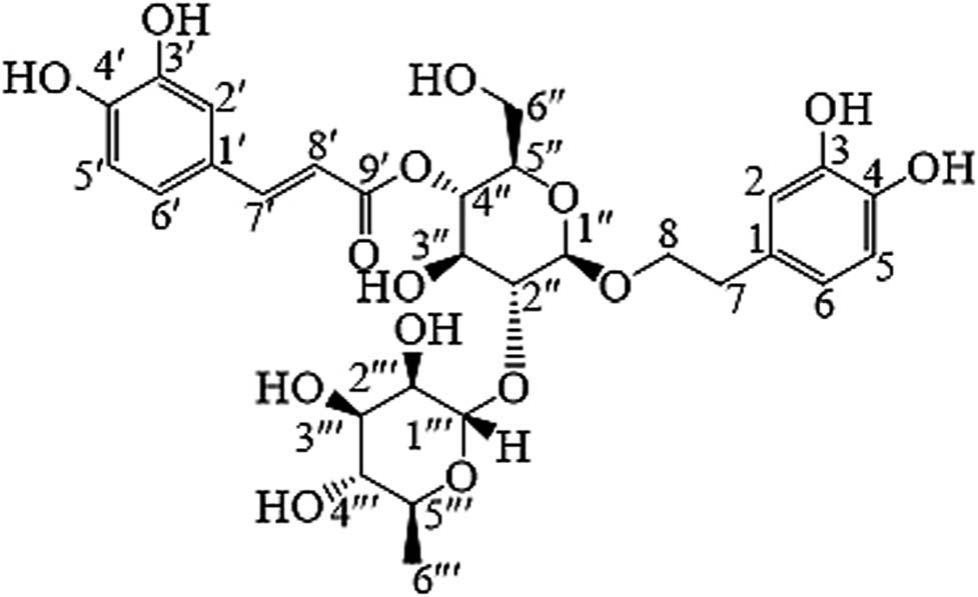
Chemical structure of acteoside (ACT).

Fraction AMPM-IV showed a major UV-active spot that gave a dark brown color when treated with anisaldehyde spray reagent followed by heating. This fraction was put onto a 3.0 × 49 cm silica gel column that was filled with hexane, and it was eluted using progressively more polar hexane/ethyl acetate solutions. Based on their TLC behavior, 40 fractions (150 mL each) were gathered and divided into 4 groups. Fraction AMPM-IV-2 showed that it contained the major interesting spots. AMPM-IV-3-4 was purified on sephadex LH-20 column (2.0 × 20 cm) using methanol as eluent. 30 Fractions were obtained and divided into 5 groups according to their TLC behavior. AMPM-IV-1-2-2 showed pure UV-active spot. After evaporation of the solvent a brown gummy material was obtained and identified as ACT (Fig. 1).

IR (KBr) *ν*_max_ cm^−1^: 3335, 3075, 1661, 1629, 1548, 1520, 1436, 1349. HRESIMS *m*/*z* 623.19864 [M − H] (calcd for [C_29_H_36_O_15_] 624.2054) ^1^H-NMR (MeOH-d_4_) *δ* 1.11(3H, d, *J* = 6.0 Hz, H6′′), 2.81(2H, m, H7), 3.32(1H, t, *J* = 9.2 Hz, H4′′), 3.41(1H, m, H2′′′), 3.53(1H, m, H5′′′), 3.56 (1H, m, H5′′), 3.59 (1H, m, H3′′), 3.62 (2H, m, H6′′′), 3.64 (1H, t, *J* = 8.8 Hz, H3′′′), 3.84 (1H, m, H2′′), 3.96 (2H, m, H8), 4.40(1H, d, *J* = 7.9 Hz, H1′′′), 4.91 (1H, bs, H4′′′), 5.21 (1H, d, *J* = 1.4 Hz, H1′′), 6.30 (1H, d, *J* = 15.9 Hz, H7′), 6.59 (1H, d, *J* = 7.8, 2.0 Hz, H6), 6.69 (1H, d, *J* = 7.8 Hz, H5), 6.71 (1H, d, *J* = 2.0 Hz, H2), 6.82 (1H, d, *J* = 8.0 Hz, H5′), 6.98 (1H, d, *J* = 7.8, 2.0 Hz, H6′), 7.09 (1H, d, *J* = 2.0 Hz, H2′), 7.61 (1H, d, *J* = 15.8 Hz, H8′). ^13^C-NMR, (MeOH-d_4_), *δ* 18.6 (C6′′), 36.5 (C7), 62.4 (C6′′′), 70.5 (C5′′), 70.9 (C4′′′), 71.0 (C3′′), 72.3 (C8), 72.4 (C2′′), 74.5 (C4′′), 74.8 (C2′′′), 78.1 (C5′′′), 80.4 (C3′′′), 101.7 (C1′′), 102.7 (C1′′′), 114.7 (C7′), 115.3 (C2′), 116.4 (C2), 116.6 (C5′), 117.2 (C5), 121.4 (C6), 123.4 (C6′), 126.3 (C1′), 130.1 (C1), 143.2 (C4), 144.7 (C3), 145.4 (C3′), 146.7 (C4′), 148.4 (C8′), 167.0 (C9′).

### 2.4. Samples preparation and weight loss measurements

The efficacy of the inhibitor was assessed using the gravimetric method using a methanolic solution of ACT acting as a copper inhibitor in an aqueous solution of 1.0 HNO_3_. Copper specimens (dimensions: 3.0 cm × 1.0 cm × 0.50 mm) that had been weighed and immersed for 2, 4, 6, and 8 hours at 28 °C in 15 mL of 1.0 M HNO_3_ solutions at various inhibitor concentrations (ACT) (100–500 mg L^−1^) were used in the weight loss experiments. Following testing, they were dried, reweighed, and thoroughly cleaned with deionized water. A water thermostat set to 0.5 °C was used for the tests, and an electronic semi-micro balance Sartorius 2024 MP6 was used to weigh the samples at 28, 33, 38, 43, and 48 °C with a precision of 0.01 mg. Three trials of the results were calculated.

### 2.5. Electrochemical measurements

To investigate corrosion behavior, two electrochemical methods were employed: electrochemical impedance spectroscopy (EIS) and Tafel polarization. For all electrochemical tests, a CorrTest potentiostat (CS350, China) was used. Copper served as the working electrode, an Ag/AgCl reference electrode, and a platinum counter electrode made up the cell. In the potential range of ±250 mV, potentiodynamic polarization measurements were performed at a scan rate of 1 mV s^−1^. To determine the corrosion current density, the linear Tafel segments were extrapolated to the corrosion potential. EIS measurements were carried out utilizing an AC signal with an amplitude of 0.1 V for the frequency spectrum between 104 Hz and 0.1 Hz in the potential range of ±200 mV. CorrTest software was used to examine all electrochemical results. To ensure that the results could be replicated, each electrochemical measurement was performed three times.

### 2.6. Surface analysis

For four hours, the copper specimens were submerged in 1.0 HNO_3_ both with and without the ideal ACT concentration. The specimens were removed after four hours, and the scanning electron microscopy (SEM) method was used to assess their surface appearance. The high energy disperses spectrometer (EDS) technology was used to identify the presence of surface components. Scanning electron microscopy (SEM) with an attached energy dispersive spectrometer (EDS) device (Thermo Scientific Phenom Desktop SEM, JU-24112022, Waltham, MA, USA) was used to conduct the SEM and EDS analysis.

### 2.7. DFT computation technique

In order to investigate the electronic properties of the ACT compound being analyzed, this study employed molecular computations based on Density Functional Theory (DFT). The calculations were performed using a 6-31G*(d,p) basis set and the B3LYP functional, both in the gas phase and in an acidic solution. Molecular structures were optimized using Gaussian 09 (G09) software, which incorporates DFT-B3LYP functionalities.^33^

The quantum chemical parameters calculated encompassed various descriptors, including the energy gap (Δ*E*_gap_) between the highest occupied molecular orbital energy (*E*_HOMO_) and the lowest unoccupied molecular orbital energy (*E*_LUMO_), the fraction of electron transferred (Δ*N*), global softness (*σ*), absolute hardness (*η*), electrophilicity index (*ω*), and chemical potential (*χ*), among others.^33,34^ These computed parameters provided significant insights into the electronic configuration and chemical stability of the compounds under investigation.

The comprehensive computational methods utilized in this study offer a deeper understanding of the chemical characteristics of the compounds under scrutiny and their potential applications. Furthermore, these computational details help elucidate the properties and possible reactivity of the substances under investigation.

## 3. Results and discussion

### 3.1. Weight loss measurements

**Fig. 2:**
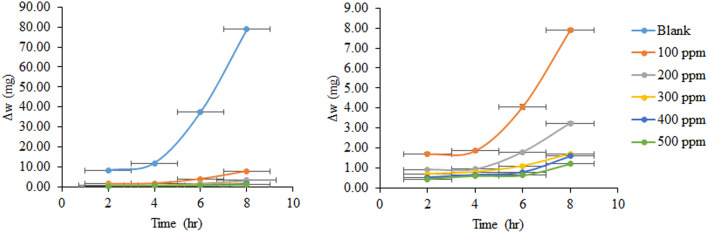
Weight loss–time curves for copper corrosion in 1.0 M HNO_3_ at 28 °C with and without varying ACT concentrations.

The weight loss data for copper in 1 M HNO_3_ with and without different ACT concentrations are shown in Fig. (2). As the inhibitor's concentration rose over time, Fig. 2 shows how the weight loss values (mg) of Cu reduced; in other words, the ACT concentration enhances corrosion inhibition. This response is expected since the copper surface is effectively separated from the medium by the metal's increased surface coverage as the ACT concentration rises.^35–39^ The inhibitory efficiency (%IE) and surface coverage (*θ*) were computed by using this equation.1
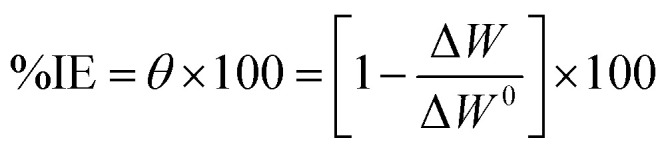


The calculated inhibition efficiency (%IE) values are shown in Table 2. From these tables, the %IE rises steadily as the inhibitor concentration rises and as the temperature rises from 28 to 48 °C. A maximum inhibition efficiency of 98.8% for 500 mg L^−1^ of ACT in acidic medium was noted at 48 °C. The compound's increased coverage of the metal surface area is most likely the reason for this behavior. It is widely known from the literature that a decrease in inhibitory efficacy with rising temperature frequently indicates the establishment of an electrostatic or physical adsorption layer. When an inhibitor is present, the opposing action (in our case) enhances inhibition efficiency as temperature rises, indicating a chemisorption process.^4–9^

**Table 2:** Corrosion parameters obtained from weight loss measurements for copper in 1.0 M HNO_3_ containing various concentrations of ACT at different temperatures

[ACT] (ppm)	Weight loss (mg)					Corrosion rate (mg h^−1^ cm^−2^)					Inhibition efficiency (%IE)				
	301 K	306 K	311 K	316 K	321 K	301 K	306 K	311 K	316 K	321 K	301 K	306 K	311 K	316 K	321 K
0	11.86	41.3	60.76	115	158.86	0.494	1.721	2.532	4.792	6.619					
100	1.86	2.4	2.7	2.8	3.03	0.078	0.083	0.113	0.117	0.126	84.3	94.2	95.6	97.6	98.1
200	0.95	1.9	2.1	2.36	2.43	0.04	0.079	0.088	0.098	0.101	92.0	95.4	96.5	97.9	98.5
300	0.8	1.4	1.7	1.76	2.13	0.033	0.058	0.071	0.073	0.089	93.3	96.6	97.2	98.5	98.7
400	0.65	1.26	1.6	1.66	1.9	0.027	0.053	0.067	0.069	0.079	94.5	96.9	97.4	98.6	98.8
500	0.6	0.93	1.4	1.56	1.83	0.025	0.039	0.058	0.065	0.076	94.9	97.7	97.7	98.6	98.8

The weight loss method was carried out at different temperatures (28 °C–48 °C) in the presence of different concentrations of ACT. Table 2 provides a summary of the weight loss, corrosion rates and inhibition efficiency of Cu in 1.0 M HNO_3_ acid as a function of temperature in the presence and absence of various inhibitor concentrations. From 28 to 48 °C, the corrosion rate of copper increased sharply in the absence of an inhibitor (ACT), but it increased more slowly when an inhibitor was included. Increasing the temperature typically speeds up the corrosion process, which in turn speeds up the metal's rate of dissolution.

Table 3 summarizes the corrosion parameter in the 28–48 °C temperature range with and without inhibitor (ACT). Using the Arrhenius equation, the activation energy (*E*_a_) for copper dissolution in 1 M HNO_3_ was determined from the slope of plots:2
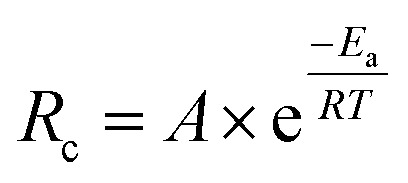
where *E*_a_ is the apparent activation energy, *A* is the constant frequency factor, and *R*_c_ is the corrosion rate.

**Table 3:** The Arrhenius and transition state activation parameters for the rate of copper corrosion in a 1.0 M HNO_3_ solution with different ACT concentrations at 28 °C

[ACT] (ppm)	*E*_a_ (kJ mol^−1^)	ΔS* (kJ mol^−1^ K^−1^)	ΔH* (kJ mol^−1^)
0	100.24	75.73	97.66
100	18.27	−213.81	15.68
200	34.00	−165.80	31.42
300	35.38	−163.15	32.79
400	39.22	−151.69	36.64
500	44.36	−136.14	41.78

A plot of log(*R*_c_) *versus* 1/*T* gives a straight line according to this equation. The values of *E*_a_ were calculated and summarized in (Table 3). In contrast to inhibited solutions, uninhibited solutions have higher energy activation. The value of the activation energy, however, rises as inhibitor concentration does. A decline in inhibition efficiency with rising temperatures and a corresponding increase in corrosion activation energy when an inhibitor is present are frequently interpreted as indicators of the development of a physical (electrostatic) adsorption coating.^8^ The result, which is consistent with a decrease in activation energy in the presence of an inhibitor and an increase in inhibition efficiency with a rise in temperature, points to a chemisorption mechanism.^35^ From the trend for the inhibitor suggests that the main effect of inhibiting species' physical adsorption (electrostatic interaction) in 1.0 M HNO_3_ is predominant.

The spontaneity of metal conversion into corrosion products is largely determined by the thermodynamic properties of the corrosion process. The transition state equation, a different formulation of the Arrhenius equation, was used to calculate the thermodynamic parameters of activation for the corrosion process (enthalpy (ΔH*)), free energy (Δ*G*), and entropy (ΔS*):3
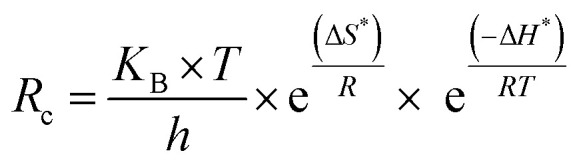
where *h* is Planck's constant, *K*_B_ Boltzmann constant, ΔS* is the entropy of activation and ΔH* is the enthalpy of activation.

According to this equation, a plot of log(*R*_c_/*T*) against (1/*T*) gives a straight line, from which the values of *E*_a_, ΔH* and ΔS* are calculated and listed in Table 2.

The activation energy (*E*_a_) and enthalpy of activation (ΔH*) for the corrosion of copper in 1 M HNO_3_ is equal to 100.24 kJ mol^−1^ and 97.66 kJ mol^−1^, respectively, which is in good agreement with the work of Zarrouk *et al.* in which he found that the activation energy and enthalpy of activation of copper in 2 M HNO_3_ is equal to 100.21 kJ mol^−1^ and 97.53 kJ mol^−1^, respectively.^35^

The literature distinguishes three types of inhibitors based on how temperature affects inhibition effectiveness:^36,37^ inhibitors whose effectiveness decreases with increasing temperature: inhibitors exhibiting a rise in inhibition efficiency as the temperature rises: *E*_a_ (uninhibited) < *E*_a_ (inhibited) *E*_a_ (uninhibited) > *E*_a_ (inhibited); inhibitors are not affected by temperature fluctuations in terms of inhibition efficiency: the presence or absence of the inhibitor has no effect on *E*_a_. Nevertheless, the inhibited solutions' ΔH* values in this investigation are lower than those of the uninhibited solutions, and they then steadily rise as the inhibitor's concentration rises, suggesting that both chemical and physical adsorption are responsible for the inhibitory action in solutions.^36,37^ This more likely indicates comprehensive adsorption, which involves both chemical and physical adsorption. Physical adsorption (electrostatic interaction) may accompany chemical adsorption since the adsorption heat was close to the overall chemical reaction heat.

The endothermic character of the metal dissolving process is reflected in the positive values of ΔH* (15.68–41.78 kJ mol^−1^). The decrease in Cu corrosion rate is mostly governed by the kinetic parameters of activation, as seen by the increase in ΔH* with increasing inhibitor concentration.^37^

In both the absence and presence of an inhibitor, the activation entropy (ΔS*) is negative. This suggests that an association rather than a dissociation occurs between reactants and the activated complex in the rate-determining step, which results in a reduction in disordering.^38^ It is clear that the ΔS* swings to less negative values (less ordered behavior) as inhibition efficiency rises.

### 3.2. Adsorption behavior

The adsorption isotherm can be used to determine whether adsorption on metallic surfaces is the primary cause of the inhibitor effect. The ACT compound's inhibitory impact on acidic corrosion of copper may be due to its adsorption onto the copper surface. By acting as a barrier between the metal surface and the aggressive solution, the adsorbed layer lowers the rate of corrosion. The following formula is used to determine the amounts of inhibitors adsorbed from the solution, or *Q*_ads_, on copper specimens when inhibitors are present:4
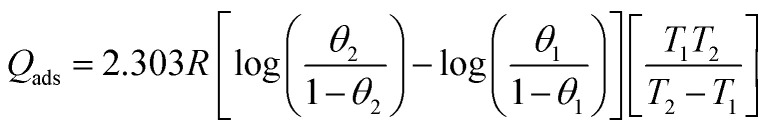
where *θ*_1_ and *θ*_2_ represent the degree of surface covering at temperatures *T*_1_ (311 K) and *T*_2_ (321 K), respectively, and *R* is the gas constant.

For acidic media, the computed *Q*_ad_ values are found to range between 49.06 and 99.26 kJ mol^−1^. An increase in efficiency at high temperatures is indicated by the positive sign of *Q*_ads_.^39^

**Fig. 3:**
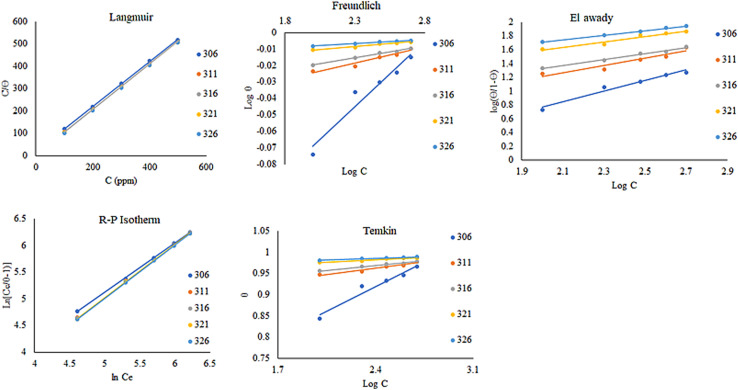
Adsorption isotherms for copper specimen with ACT in 1.0 M HNO_3_ for 4 h at different temperatures.

The corrosion inhibition in the current study was better understood thanks to the isotherms used to illustrate the process of crude adsorption on the copper surface. The type of copper metal, the compound's interaction with the copper surface, the size and structure of the organic compound, and the adsorption mechanism are some of the variables that affect how effective ACT is as a corrosion inhibitor. The most widely used adsorption isotherm systems, including the El-Awady, Freundlich, Flory–Huggins, Langmuir, Redlich–Peterson (R–P), Dubinin–Radushkevich, Temkim, and Frumkin isotherm models, were used to test adsorption modes in order to better understand the mechanism of corrosion inhibitor and the adsorption behavior of ACT on copper surface. Based on correlation (*R*^2^) values, which should be near unity, the adsorption process tends to follow the Langmuir, Freundlich, El-Awady, Temkim, and Redlich–Peterson (R–P) adsorption isotherms (Fig. 3). Table 3 lists the adsorption parameters that were thus determined. The Redlich–Peterson (R–P) isotherm provided the greatest match, followed by the Langmuir, El-Awady, Freundlich, and Temkin isotherms, according to the regression coefficient values shown in Table 4. Data from weight loss measures best fit the Langmuir adsorption isotherm, according to attempts to fit the data into several adsorption isotherms. According to Langmuir's assumptions, the degree of surface covering (*θ*) and the concentration of the adsorbate in the electrolyte's bulk (*C*_inh_) are related in *E*_q_.5
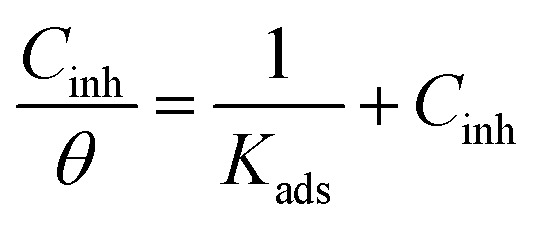
where *C*_inh_ is the concentration of the inhibitor and *K*_ads_ is the equilibrium constant of the adsorption–desorption process. The association between *C*_inh_/*θ* and *C*_inh_ at the optimal inhibitor concentration is seen in Fig. 3 as a straight line. This outcome demonstrates that the slope is quite near to unity and the regression coefficient (*R*^2^) is nearly equal to unity. This suggests that the Langmuir adsorption isotherm governs the adsorption of ACT. Since the Langmuir-type adsorption isotherm in 1.0 M HNO_3_ had a slope of nearly unity, the inhibitor species' monolayer must have adhered to the Cu surface without any lateral interactions amongst the adsorbed species.

**Table 4:** Adsorption values obtained for copper specimen in ACT in 1.0 M HNO_3_ for 4 h at different temperatures

Adsorption isotherm		306 K	311 K	316 K	321 K	326 K
Langmuir adsorption isotherm	*R* ^2^	0.9999	0.9999	1.0000	1.0000	1.0000
	Slope	1.0219	1.0147	1.0171	1.0103	1.0093
	Intercept	14.9510	5.5628	3.4008	1.7237	1.1480
	*K*_ads_	0.0669	0.1798	0.2940	0.5802	0.8710
	Δ*G*_ads_ (kJ mol^−1^)	−27.71	−31.29	−33.09	−35.42	−37.08
	RL	0.1357	0.0263	0.0111	0.0043	0.0023
Freundlich adsorption isotherm	*R* ^2^	0.9778	0.9575	0.9909	0.9663	0.9945
	Slope	0.0807	0.0197	0.0141	0.0074	0.0050
	Intercept	−0.2757	−0.0642	−0.0477	−0.0255	−0.0182
	*K*_f_	0.5301	0.8627	0.8959	0.9430	0.9589
	1/*n*	0.0807	0.0197	0.0141	0.0074	0.0050
	Δ*G*_ads_ (kJ mol^−1^)	−33.81	−35.35	−36.01	−36.72	−37.34
El-Awady adsorption isotherm	*R* ^2^	0.9671	0.9108	0.9863	0.9620	0.9984
	Slope	0.7722	0.5306	0.4320	0.4000	0.3337
	Intercept	−0.7771	0.1503	0.4626	0.7921	1.0428
	1/*y*	1.30	1.88	2.32	2.50	3.00
	*K*_ads_	0.10	1.92	11.78	95.59	1.33 × 10^3^
	Δ*G*_ads_ (kJ mol^−1^)	−30.60	−36.62	−39.10	−41.75	−43.96
Redlich–Peterson (R–P) isotherm	*R* ^2^	0.9994	1.0000	1.0000	1.0000	1.0000
	Slope	1.0731	1.0152	1.0094	1.0025	1.0001
	Intercept	−0.4948	−0.1188	−0.0797	−0.0271	−0.0101
	*α*_R_	0.6097	0.8880	0.9234	0.9732	0.9899
	*β*	1.0731	1.0152	1.0094	1.0025	1.0001
	*K*_R_	1	1	1	1	1

Ideal simulating 1 is indicated by the slope of the *C*_inh_/*θ vs. C*_inh_ plots being unity, as predicted by the Langmuir adsorption isotherm. One possible explanation is the interactions between the adsorbed species on the copper surface.^1,36^ The free energy of adsorption (Δ*G*_ads_) and adsorption equilibrium constant (*K*_ads_) were calculated and given in Table 4 using the following relationship.

6 Δ*G*^0^_ads_ = −*R* × *T* × Ln(55.5 × *K*_ads_)

where *R* is the universal gas constant and 55.5 is the water in solution concentration in mol L^−1^.

In general, electrostatic interactions are consistent with Δ*G*_ads_ values of −20 kJ mol^−1^ or less, while chemical interactions are defined as those with values of −40 kJ mol^−1^ or greater.^1,40^ In this investigation, the computed Δ*G*_ads_ values in an acidic medium ranged from −3.34 to −10.51 kJ mol^−1^ as the temperature rose from 28 to 48 °C. This suggests that the inhibitor's adsorption on the copper surface occurs spontaneously and validates the physical adsorption mechanism.

Additionally, the value of *K*_ads_ has been observed to rise with temperature, suggesting that the adsorption of inhibitor molecules on the copper surface was more advantageous at higher temperatures. These findings suggest that, in this investigation, the interactions between the adsorbed molecules and the metal surface intensify as the temperature rises to 48 °C. This finding explains why the inhibition efficiency rises as the temperature rises. Nonetheless, the modest reported values for the free energy shift Δ*G*_ads_ validate the tested ACT inhibitor's physisorption action on the copper surface.

**Fig. 4:**
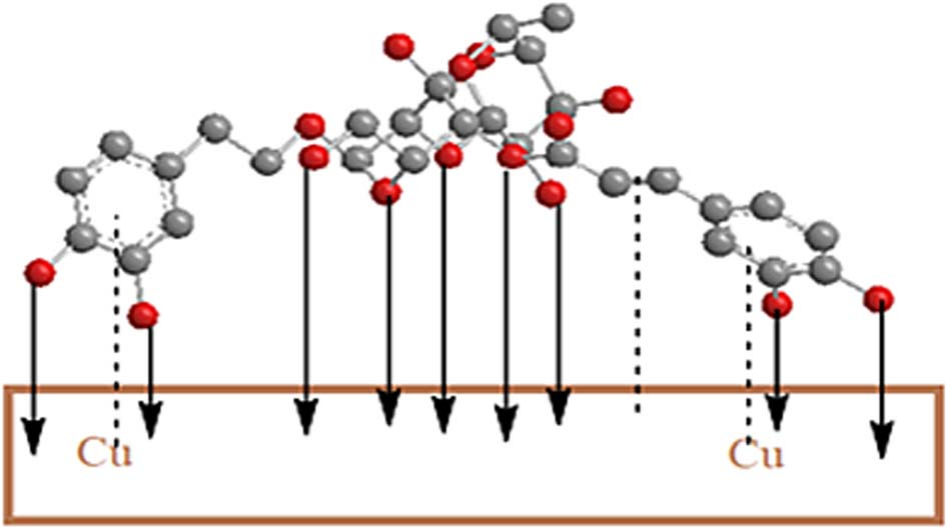
Schematic illustration of inhibition mechanism of ACT adsorption on copper in 1.0 M HNO_3_ yielding the formation of protective layer.

The equilibrium parameter *R*_L_, also known as the separation factor or equilibrium parameter, is a dimensionless constant that can be used to express the essential characteristics of the Langmuir isotherm.^41–43^7
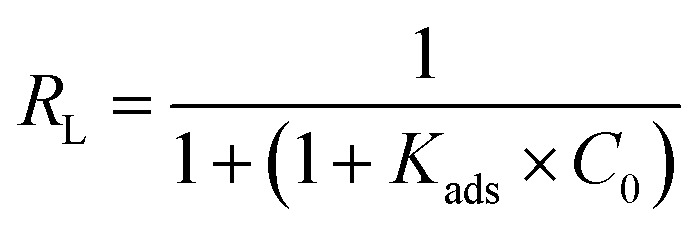
where: *C*_0_ = initial concentration *K*_ads_ = the constant related to the energy of adsorption (Langmuir constant). Adsorption is said to be unfavorable if *R*_L_ > 1, linear if *R*_L_ = 1, and favorable if *R*_L_ < 0.^41–43^ According to the calculated value of *R*_L_, the molecules of the ACT compound that are adsorbed on the copper's surface are in good shape (Fig. 4).

El-Awady isotherm also best fits the experimental data.^33^ This equation describes the El-Awady isotherm.8
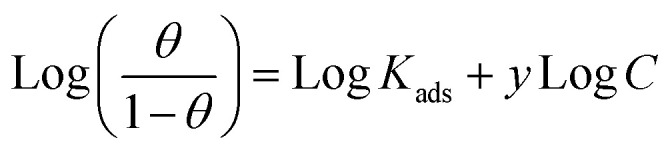
where *K*_ads_ is a constant related to the adsorptive equilibrium constant, *θ* is surface coverage, *C* is concentration, and *y* is the number of inhibitor molecules that can fit in a single active site.9
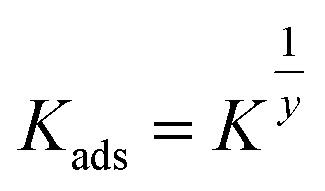


If 1/*y* is less than one, the inhibitor is likely to form numerous layers on the metal surface; if 1/*y* is more than one, the inhibitor molecule is likely to occupy many active sites.^42–44^ Each inhibitory molecule was attached to several active sites on the copper surface, as evidenced by the current values of 1/*y* being greater than one. It might be because the compound hydrolyzed in the presence of HNO_3_ solution, resulting in the presence of more than one anodic site with the compound.

The Freundlich isotherm describes the degree of adsorbent surface heterogeneity in multilayer adsorption.^42,43^ The linear form of the Freundlich isotherm equation is provided by the following formula.10
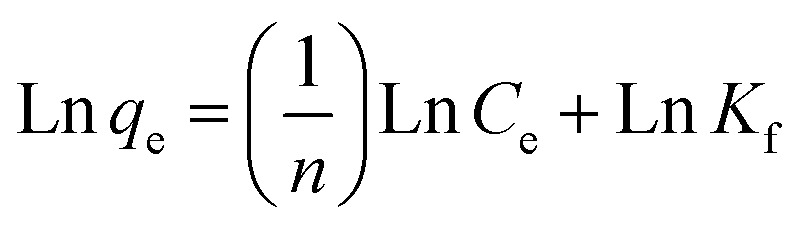


The intensity and capacity of adsorption are related by the Freundlich isotherm constants *K*_f_ (L g^−1^) and 1/*n* (dimensionless), respectively. The constants 1/*n* and *K*_f_ are determined using the slope and intercept of the linear plot of lnqe against ln *C*_e_ shown in Fig. 3. Table 4 displays the Freundlich isotherm constants. According to the *R*^2^ score, this isotherm fit the experimental data quite well. The ease of adsorption is described by the value of 1/*n*. Adsorption is typically thought to be easy when 0 < 1/*n* < 1 and moderate or challenging when 1/*n* = 1 or 1/*n* > 1, respectively.^44^ An effective physical adsorption process between copper ions and ACT is indicated by the obtained 1/*n* value, which is again smaller than unity.

The Redlich–Peterson (R–P) isotherm is quite versatile and can be applied to both homogeneous and heterogeneous systems. When compared to the Langmuir, Freundlich, and D–R isotherms, the R–P isotherm incorporates three parameters into a single equation.^43^ This is the linear form of the R–P isotherm.11
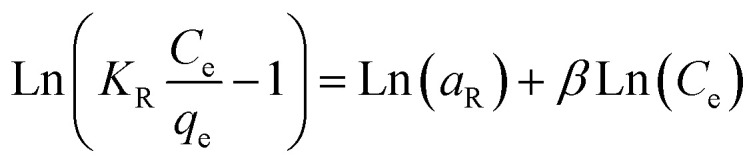
where the R–P isotherm constants are *K*_R_ (L g^−1^) and *α*_R_ (L mg^−1^) 1/*β*, and the exponent, *β*, is between 0 and 1. The R–P isotherm approximates Henry's law at low concentrations and approaches the Freundlich isotherm at large concentrations. The use of linear regression analysis is more complicated because this isotherm takes into account three parameters. For the linear plot of ln[*K*_R_*C*_e_/*q*_e_ − 1] against ln *C*_e_, this isotherm was applied by adjusting the isotherm parameter *K*_R_ in order to maximize the regression *R*^2^. The constants *β* and *α*_R_ were derived from the slope and intercepted, respectively, of this plot, which is displayed in Fig. 3. Table 4 records regression *R*^2^ as well as the *K*_R_, *α*_R_, and *β* values.

Another well-known adsorption isotherm that is frequently used to explain how corrosion inhibitors work is the Temkin model. Unlike those covered thus far, it provides some information about the kind of interactions taking place in the adsorbed layer. The model is expressed as follows:

12 e^2*aθ*^ = *K* × *C*_e_

The sign of the molecular interaction parameter (*α*) is utilized to establish whether repulsion or attraction takes place in the adsorbed layer.^43,45^ The linearized form of the equation can be used to create linear plots of *θ* against ln *C* (Fig. 4).13
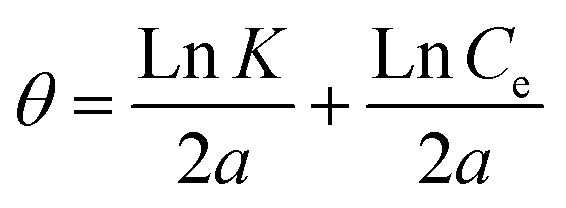


The strength of the inhibitor molecules' adsorption on the metal surface is indicated by the value of *K*.

### 3.3. Potentiostatic polarization measurements

**Fig. 5:**
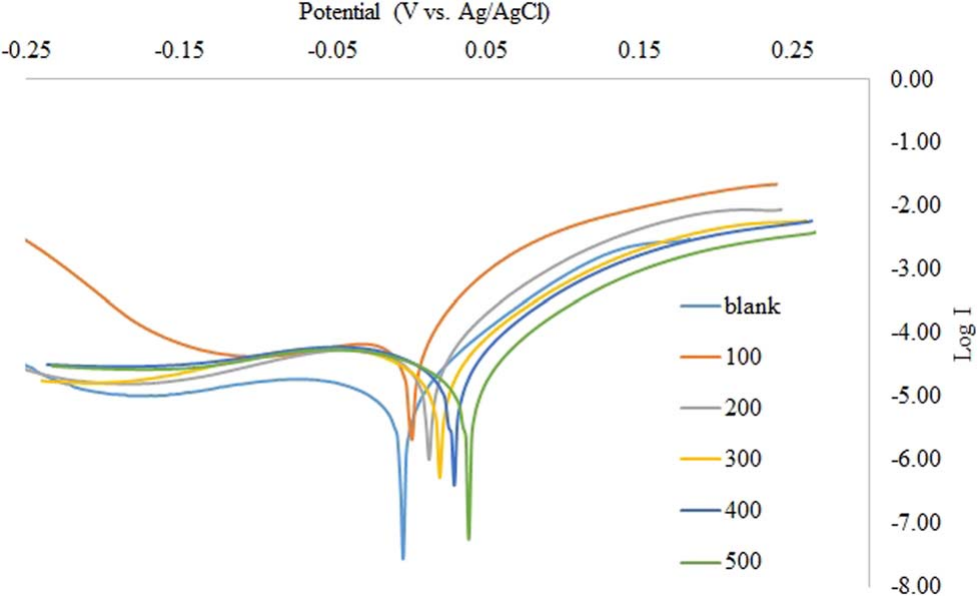
Copper polarization anodic and cathodic curves in 1.0 M HNO_3_ with and without different ACT concentrations.

For HNO_3_, the polarized anodic and cathodic potentials were observed with and without varying inhibitor (ACT) concentrations in a current density range of 0.7–7 A cm^−2^. Fig. 5 displays the copper cathodic and anodic polarization curves in 1.0 M HNO_3_ with and without varying inhibitor (ACT) concentrations.

The shape of the polarization curve (Fig. 5) makes it evident that anodic and cathodic reactions are inhibited. They are evident from the data in Table 5 that as the concentration of the inhibitor (ACT) increases, the values of the anodic (*β*_a_) and cathodic (*β*_c_) Tafel constants also alter. This bolsters the mixed mode (cathodic and anodic) inhibitory activity of the acteoside molecule.

**Table 5:** Copper's electrochemical corrosion characteristics in 1.0 M HNO_3_ with and without different inhibitor (ACT) concentrations

[Inhibitor] (ppm)	*E*_corr_ (mV s^−1^) (Ag/AgCl)	*I*_corr_ (mA cm^−2^)	*R*_p_ (Ω cm^2^)	*β*_a_ (mV dec^−1^)	*β*_c_ (mV dec^−1^)	%IE
Blank	−4.23	0.697	29.80	188.89	64.00	
100	1.75	0.266	54.33	60.92	73.37	61.8
200	12.70	0.210	111.43	71.26	221.26	69.8
300	19.60	0.126	230.46	72.48	824.33	82.0
400	29.10	0.103	296.20	75.34	1033.16	85.2
500	38.60	0.091	356.88	80.11	1095.29	87.0

Copper's corrosion potential (*E*_corr_), corrosion current density (*I*_corr_), anodic Tafel constant (*β*_a_), cathodic Tafel constant (*β*_c_), and inhibition efficiency (%IE) were all determined using the values of its polarization curves in 1.0 M of HNO_3_ with and without different inhibitor concentrations. Table 4 then provides a summary of this data.

In specimens containing 500 ppm of ACT at 25 °C, the value of *β*_c_ > *β*_a_. The anode ion breaks down into the HNO_3_ solution when the value of *β*_c_ > *β*_a_. In contrast, the values of *β*_c_ < *β*_a_ for the remaining instances. The absence of metal ion breakdown at the anode is indicated by a value of *β*_c_ < *β*_a_.^8^

Table 5 shows that the corrosion current density (*I*_corr_) values decrease from 0.697 mA cm^−2^ to 0.091 mA cm^−2^ when different concentrations of ACT are added, demonstrating that ACT has a significant inhibitory effect on the corrosion of copper in these media. The %IE increases from 61.8% to 87.0% as the *I*_corr_ decreases. At a concentration of 500 ppm inhibitor, the maximum %IE was reached at 87.0%. Generally speaking, if a compound's displacement in *E*_corr_ is greater than 85 mV relative to the *E*_corr_ of the blank, it can be classified as either an anodic or cathodic kind of inhibitor; if it is less than 85, it can be considered a mixed type.^8^ The maximum displacement in our study was 38.60 mV, indicating that the ACT is a mixed-type inhibitor. Additionally, it is discovered that *R*_p_ value rises as inhibitor concentration is increased, suggesting that copper corrosion is slowed down in inhibited solution *versus* uninhibited. When the inhibitor concentration grew from 100 ppm to 500 ppm, the maximum %IE determined from the Tafel polarization ranged between 61.8% and 87.0%. However, the %IE attained with weight loss (79.5–94.6%) is a little bit greater than that attained through electrochemical investigations. While the stabilization period for electrochemical measurements is only 30 min, the 2 h immersion time in weight loss measures may be the cause of this difference.

The acteoside (ACT) compound offers very good protection to copper against corrosion in acidic media, according to the results of both weight loss and polarization procedures. This is explained by the acteoside film's relative stability, which developed on copper's surface.

### 3.4. Electrochemical impedance spectroscopy (EIS)

**Fig. 6:**
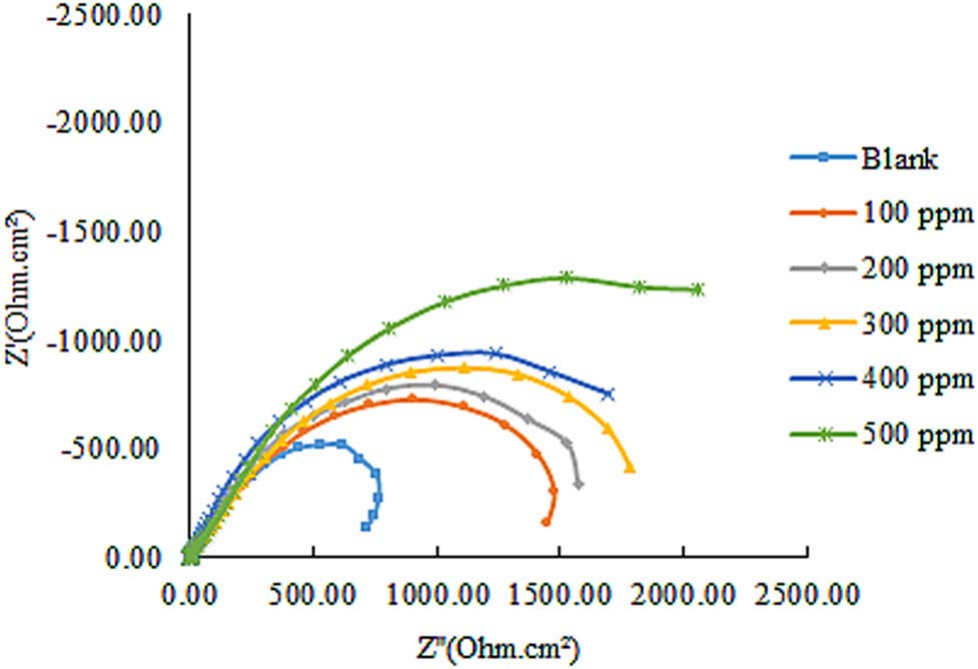
Nyquist plots for Cu corrosion at 303 K using the investigated inhibitor ACT and in a 1.0 M HNO_3_ solution.

**Fig. 7:**
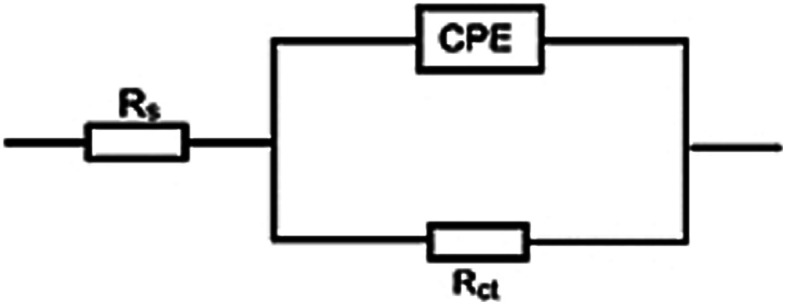
The EIS data for Cu corrosion in 1.0 M HNO_3_ solution and with the investigated inhibitor ACT were fitted using an electrochemical equivalent circuit.

Fig. 6's Nyquist plots showed that all systems had semicircles over the frequency range under study. The semicircles' diameter grows with the inhibitor concentration and is greater in the presence of ACT than in the blank solution. This suggests that as the inhibitor concentration rises, so does the inhibited substrate's impedance. The solitary charge transfer step during the dissolution reaction is depicted by a single semicircle in the picture. The impedance spectrum was analyzed by fitting information to the equivalent circuit model (Fig. 7). According to the impedance data in Table 6, the electrochemical double layer capacitance (*C*_dl_) value decreases and the *R*_ct_ value increases with the addition of the ACT. The increase in the *R*_ct_ value is caused by the formation of the protective layer on the metal/solution contact.^46–49^ The thickness of the electric double layer increases when *C*_dl_ decreases.^50^ This result suggests that by sticking to the surface of the copper, the molecules stopped it from corroding, increasing *R*_ct_ values and lowering *C*_dl_ values.^50^

**Table 6:** Cu corrosion electrochemical impedance parameters in 1.0 M HNO_3_ solution with different ACT concentrations

Conc. (ppm)	*R*_S_, Ω cm^2^	*R*_ct_, Ω cm^2^	*C*_dl_, μF cm^−2^	CPEP	%IE
Blank	1.75	240.00	286.81	0.77	
100	1.42	1719.40	235.04	0.77	86.04
200	1.42	1973.70	221.57	0.78	87.84
300	1.31	2286.50	214.40	0.75	89.50
400	1.14	2612.20	203.87	0.78	90.81
500	1.06	3789.50	196.46	0.75	93.67

The inhibition efficiency from the impedance data was computed by comparing the charge transfer resistance values with and without the inhibitor.14
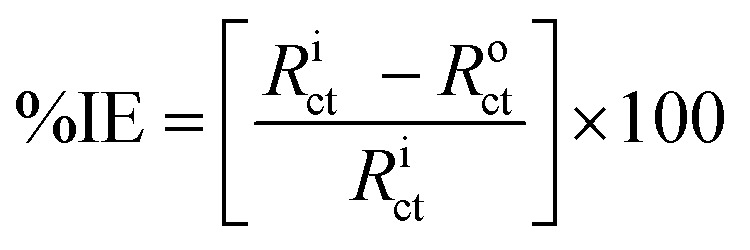
where *R*^i^_ct_ and *R*^o^_ct_ stand for the charge transfer resistance values with and without ACT, respectively. The size and trend of the obtained values displayed in Table 6 are quite similar to the values obtained from electrochemical polarization and gravimetric measurements.

### 3.5. Surface analysis

**Fig. 8:**
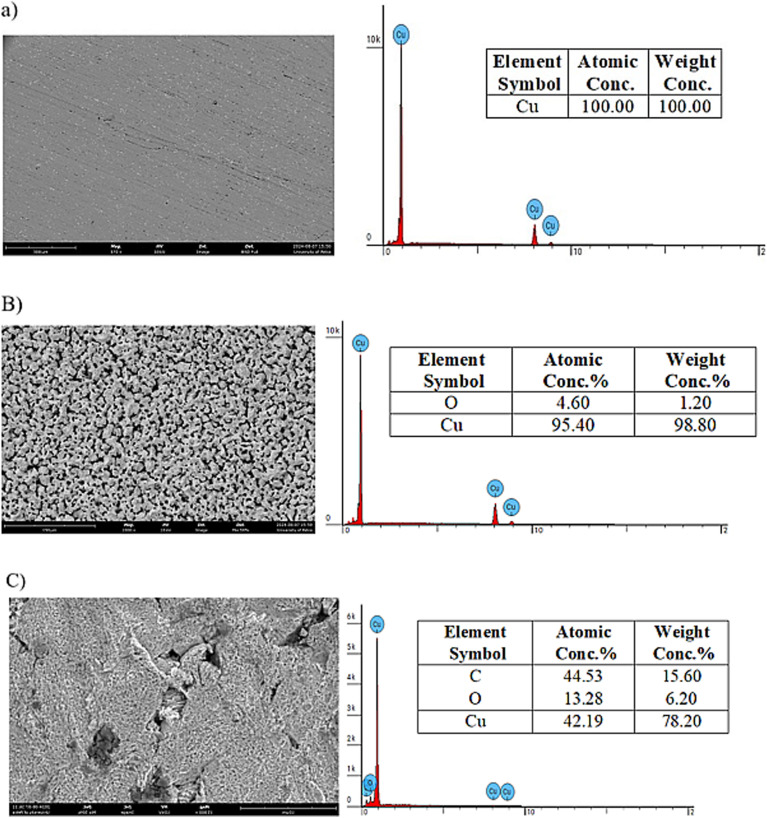
SEM image and EDS mapping of copper surface (A) without immersion (B) in 1.0 M HNO_3_ (C) presence of 300 ppm of ACT.

The surface was examined both with and without an inhibitor before and after 4 hours of immersion in 1.0 M HNO_3_. The polished surface of copper is seen in Fig. 8A before exposure to the testing environment. Fig. 8a shows the copper surface's polishing lines. The surface was shown to be pure copper specimen (Fig. 8a) and free of any corrosion products by the relative EDS graph. As a result of the corrosive medium's attack, the copper surface that was submerged in the tested medium for eight hours without inhibitors seems severely damaged and corroded. The EDS analysis shows the presence of oxygen, which is a corrosive element for copper and therefore justifies the corrosion of copper (Fig. 8b). Nevertheless, once the inhibitors were added, the copper surface's roughness improved, and it seemed to be less damaged. Deposits or adsorbates were also found on the surface, indicating that an inhibitor protective layer had formed over the copper surface (Fig. 8c). The EDS spectrum in Fig. 8a shows characteristic peaks of the copper surface elements before immersion in nitric acid, where a 100% mass fraction of copper is obtained. The intensity of the copper peak (98.80% copper) is reduced in the EDS spectrum of copper without inhibitor, and a characteristic peak intensity of oxygen appears in the range (1.20% O) compared to the raw copper, which is depicted in Fig. 8b. In the presence of ACT inhibitor, the spectrum displays the appearance of another characteristic peak for the following elements C, O and Cu with degree of 15.60%, 6.20% and 78.20% respectively, which is depicted in Fig. 8c. These findings demonstrate how the tested ACT interacts with the copper surface in an acidic solution to form a protective barrier coating.

### 3.6. Quantum chemical calculation

**Fig. 9:**
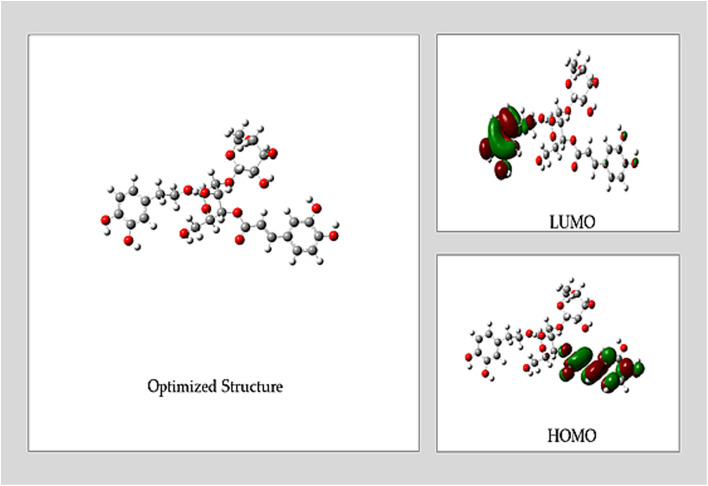
ACT in acidic media: optimized geometry, HOMO, and LUMO.

Understanding the intriguing process of molecules adhering to metal surfaces and their subsequent chemical reactivity depends on the Frontier Molecular Orbital Theory (FMO). Reactivity is mostly determined by the interaction between a molecule's Lowest Unoccupied Molecular Orbital (LUMO) and Highest Occupied Molecular Orbital (HOMO) levels.^33^ In this groundbreaking study, state-of-the-art DFT calculations were conducted on the prominent compound ACT, sourced from the ethanolic extract of the plant. The study explored both gas phase and acidic solution (HNO_3_) conditions in great detail, pushing the boundaries of traditional investigations. Fig. 9 provides a visually captivating depiction of the optimized geometry of these important compounds, revealing the unique characteristics of their molecular orbitals (HOMO and LUMO) in unprecedented detail.

This study uses a novel method to examine a molecule's reactivity to adsorption on a copper-metallic surface. The emphasis is on assessing the Δ*E*_gap_, a critical metric that represents the inhibitor's effectiveness and level of reactivity. Higher reactivity and better inhibitory performance are indicated by a decrease in the Δ*E*_gap_ value. The improved inhibitory potential that results from the complex formed between the primary organic component and the surface is the cause of this effect.^33^

The *σ* and *η* parameters, which are obtained from Δ*E*_gap_ and offer more information, are also included in the analysis. The *σ* parameter, which is associated with the polarizability of the molecule, emphasizes how responsive a softer molecule with a smaller Δ*E*_gap_ is, leading to increased reactivity.^50,51^ To fully explore these intriguing phenomena, a number of quantum chemistry and molecular dynamics parameters, such as Δ*E*_gap_, *σ*, Δ*N*, *η*, *X* and *ω*, were carefully computed for the ACT molecule. Table 7 presents the results, which provide fresh insight into the compound's adsorption behavior.

**Table 7:** Quantum parameters and calculated properties for ACT (*X*_Cu_ = 4.48 eV; *η*_Cu_ = 3.25)^50,51^

Physical state	*E*_HOMO_ (eV)	*E*_LUMO_ (eV)	Δ*E*_gap_ (eV)	*σ*	*η*	*X*	*ω*	Δ*N*	Dipole moment, *μ* (Debye)
Gas	−8.117	−5.428	2.689	0.743	1.344	6.772	7.460	0.249	3.325
Acidic	−5.508	−1.492	4.016	0.498	2.008	3.500	4.997	0.093	4.643

**Fig. 10:**
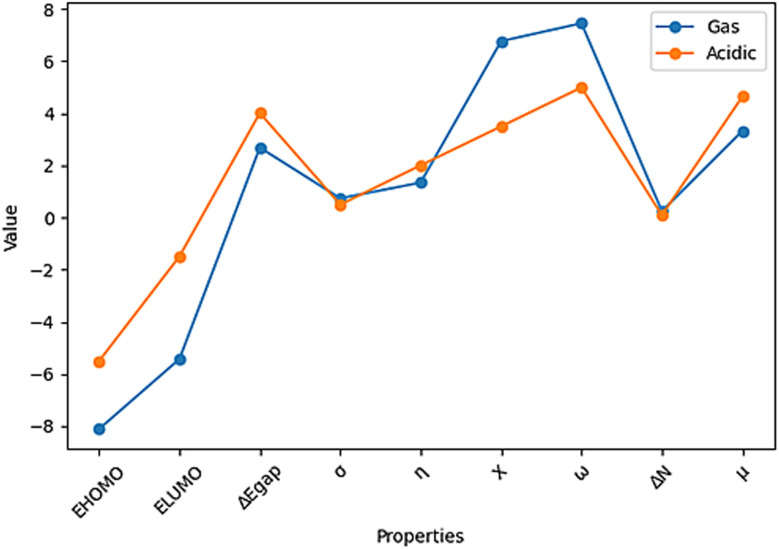
The quantum parameters and calculated properties for the ACT compound in the gas and acidic states.

Both the gas and acidic states of the ACT compound have shown promising potential in protecting copper surfaces and serving as environmentally friendly inhibitors, as shown in Table 7 and Fig. 10. DFT calculations have revealed no significant differences in the calculated quantum parameters between the two states. The Δ*E*_gap_ values of the ACT compound exhibit an increasing trend from ACT (gas) to ACT (acidic). Notably, ACT (gas) has the highest softness (*σ*) and the lowest global hardness (*η*) values. In addition, compared to the acidic media, ACT (gas) displays a higher electrophilicity index (*ω*), which is supported by its elevated *E*_LUMO_ value. The inhibitor's capacity to take up electrons from the metal surface is shown by the electrophilicity index (*ω*).^51^ The *E*_LUMO_ value (−5.428 eV) of ACT (gas) indicates its strong capacity to accept electrons from copper. Furthermore, the fraction of transferred electrons (Δ*N*) reveals that ACT (gas) contributes a higher proportion of transferred electrons to the copper surface (Δ*N* = 0.249) compared to ACT (acidic). It is worth noting that inhibitors with greater electron-donating ability at the metal surface tend to exhibit increased efficiency when Δ*N* is less than 3.6.^51^

Based on their Δ*N* values, which are less than 3.6, the compounds examined in both stages of the current investigation can be classified as green inhibitors. This chemical is the most effective inhibitor among those studied in both gaseous and acidic settings because it acts as the primary contributor to electron transfer onto the copper surface.

The presence of oxygen hetero-atoms and π-electrons in the aromatic rings of the ACT compound contributes to its adsorption in both phases.^33,52^ The results show that ACT may donate electrons from electron-rich centers to the copper's unoccupied d orbitals and accept electrons from the copper surface to establish a back-donating connection, allowing it to absorb onto the surface in all phases. The inhibitor's spatial orientation in the optimized structure determines whether this bond forms. The findings imply that the ACT molecule donates the unshared pair of electrons from oxygen atoms to the unoccupied d-orbitals of copper, hence demonstrating the strongest capacity to adsorb onto the copper surface. Consequently, it is anticipated that the ACT's adsorption on the copper surface will be the main cause of its inhibitory efficiency. In summary, physisorption and chemisorption are the two ways that ACT adsorbs on the Cu surface in acidic media. The difference between the inhibitors' and copper metal's chemical potential determines how much adsorption occurs. ACT (gas) and ACT (acidic) have computed Δ*G*_ads_ values of −52.854 and −22.599, respectively. Since the Δ*G*_ads_ values fall between zero and −40 kJ mol^−1^, they indicate that the inhibitors' adsorption mechanism is spontaneous physisorption.

## 4. Conclusions

The current investigation leads to the following key conclusions:

(1) Acteoside compound (ACT) was proposed as a green corrosion inhibitor for copper in an acidic medium (1.0 M HNO_3_) based on the results of weight loss experiments and polarization techniques.

(2) The inhibition effectiveness rose as ACT concentration was increased, reaching a maximum value at 500 ppm at 48 °C. The inhibition efficiency also increased as temperature was raised.

(3) In the absence of an inhibitor, pure copper was found to dissolve in solution with a higher activation energy (*E*_a_) than when an inhibitor was present. This implies that ACT-induced copper corrosion was successfully avoided by both the passivation procedure and barrier protection.

(4) The equilibrium isotherms for the adsorption of ACT on copper in 1.0 M HNO_3_ at different temperatures were investigated using four well-known isotherm models: the Langmuir, Freundlich, El-Awady, and Redlich–Peterson (R–P) isotherms.

(5) The sign of the free energy of adsorption indicates that the process is spontaneous, and the free Gibbs energy value showed that the physical adsorption in acidice midia could be ascribed to the inhibitory mechanism.

(6) The inhibitor (ACT) is a mixed type inhibitor (anodic and cathodic), according to polarization measurements.

(7) The ACT compound exhibited the most notable adsorption capacity on the copper surface, in both gas and acidic environments, thereby positioning it as a highly promising copper corrosion inhibitor. The adsorption mechanism was found to encompass a combination of physisorption and chemisorption. The favorable and spontaneous nature of the adsorption process is corroborated by the calculated Δ*G*_ads_ values.

(8) Potentiodynamic polarization studies reveal that ACT acts as mixed type inhibitors.

(9) The EIS analysis indicates that corrosion resistance or impedance increase with ACT by the formation of a protective layer on metal.

(10) SEM and EDS analysis show that ACT forms a protective film on the copper surface.

(11) The experimental and DFT results showcased in this study greatly augment our comprehension of the molecular interactions existing between inhibitors and metal surfaces. These findings offer invaluable insights that can be harnessed for the advancement of innovative and efficacious strategies for corrosion inhibition. Further investigation is imperative to explore the pragmatic implementation of ACT compound and other similar inhibitor in real-life scenarios of corrosion protection. This inquiry will serve to bridge the disparity between theoretical predictions and practical implementation, thereby propelling the progress of corrosion prevention techniques.

## Data availability

The data supporting this study are included within the article.

## Author contributions

Mahmoud A. Al-Qudah: conceptualization, supervision, methodology, investigation, formal analysis, data curation, funding acquisition, writing – original draft, writing – review & editing. Tareq T. Bataineh: conceptualization, methodology, formal analysis, writing – review & editing. Faten M. Abu Orabi: methodology, writing – review & editing. Sultan T. Abu-Orabi: supervision, investigation, funding acquisition, writing – review & editing. Ghassab M. Al-Mazaideh: conceptualization, methodology, investigation, formal analysis, data curation, writing – original draft, writing – review & editing. Abbas I. Alakhras: methodology, formal analysis, writing – review & editing.

## Conflicts of interest

The authors declare no conflicts of interest.

## Supplementary Material

RA-015-D5RA01657F-s001

## References

[cit1] Fateh A., Aliofkhazraei M., Rezvanian A. R. (2020). Arabian J. Chem..

[cit2] Tansuğ G. Ö., Tüken T. U., Giray E. S., Fındıkkıran G., Sığırcık G., Demirkol O. N. U. R., Erbil M. E. H. (2014). Corros. Sci..

[cit3] Elabbasy H. M. (2019). Int. J. Electrochem. Sci..

[cit4] Fouda A. S., Shalabi K., Idress A. A. (2015). Green Chem. Lett. Rev..

[cit5] El-Dossoki F. I., El-Nadr H. A., El-Hussein A. (2018). Zaštita materijala.

[cit6] Rahal C., Masmoudi M., Abdelhedi R., Sabot R., Jeannin M., Bouaziz M., Refait P. (2016). J. Electroanal. Chem..

[cit7] Alemnezhad M. M., Ghaffarinejad A., Omidali F. (2023). J. Adhes. Sci. Technol..

[cit8] Fouda A. S., Abdallah Y. M., Elawady G. Y., Ahmed R. M. (2015). J. Mater. Environ. Sci..

[cit9] Houbairi S., Lamiri A., Essahli M. (2014). Chem. Sci. Rev. Lett..

[cit10] Wedian F., Al-Qudah M. A., Al-Mazaideh G. M. (2017). Int. J. Electrochem. Sci..

[cit11] Koumya Y., Idouhli R., Zakir O., Khadiri M. E., Zaki M., El Karroumi J., Benyaich A. (2021). Chem. Pap..

[cit12] Ameer M. A., Fekry A. M. (2011). Prog. Org. Coat..

[cit13] Dharmaraj E., Pragathiswaran C., Govindhan P., Arockia Sahayaraj P., John Amalraj A., Dharmalingam V. (2017). Int. J. Res. Pharm. Chem..

[cit14] Al-Qudah M. A. (2011). J. Chem..

[cit15] Swetha G. A., Sachin H. P., Guruprasad A. M., Prasanna B. M., Sudheer Kumar K. H. (2018). J. Bio Tribo Corros..

[cit16] Sithuba T., Masia N. D., Moema J., Murulana L. C., Masuku G., Bahadur I., Kabanda M. M. (2022). Results Eng..

[cit17] Durgadevi S., Rose A. L., Vidhya S., Priya F. J., Kanmani P. L. F. (2022). Orient. J. Chem..

[cit18] Veys-Renaux D., Reguer S., Bellot-Gurlet L., Mirambet F., Rocca E. (2018). Corros. Sci..

[cit19] Samal P. P., Singh C. P., Krishnamurty S. (2023). Appl. Surf. Sci..

[cit20] Deyab M. A., Mohsen Q., Bloise E., Lazzoi M. R., Mele G. (2022). Sci. Rep..

[cit21] Al-Qudah M. A., Bataineh T. T., Alakhras A. I., Al-Mazaideh G. M. (2025). Appl. Phys. A:Mater. Sci. Process..

[cit22] Klaus J., Nassar N., Electrochem J. (2013). Sci. Technol..

[cit23] Savita S., Mourya P., Chaubey N., Singh V. K., Singh M. M. (2016). Metall. Mater. Trans. B.

[cit24] Houbairi S., Essahli M., Lamiri A. (2014). Int. J. Eng. Res. Technol..

[cit25] Al Jahdaly B. A. (2023). Arabian J. Chem..

[cit26] Barouni K., Kassale A., Bazzi L., Salghi R., Hammouti B., Albourine A., El Issami S., Jbara O., Bouachrine M. (2014). Res. Chem. Intermed..

[cit27] Barouni K., Kassale A., Albourine A., Jbara O., Hammouti B., Bazzi L. (2014). J. Mater. Environ. Sci..

[cit28] Finšgar M., Milošev I. (2010). Corros. Sci..

[cit29] Chiter F., Costa D., Maurice V., Marcus P. (2021). npj Mater. Degrad..

[cit30] Madkour L. H., Elshamy I. H. (2016). Int. J. Ind. Chem..

[cit31] El-Asri A., Jmiai A., Lin Y., Taoufyq A., Rguiti M. M., Bourzi H., El Issami S. (2022). Corros. Eng., Sci. Technol..

[cit32] M Al-Mazaideh G. (2024). Global NEST J..

[cit33] Al-Mazaideh G. M., Abu-Sbeih K. A. A., Khalil S. M. (2017). J. Chem., Biol. Phys. Sci..

[cit34] Al-Mazaideh G. M. (2022). Int. J. Corros. Scale Inhib..

[cit35] Zarrouk A., Warad I., Hammouti B., Dafali A., Al-Deyab S. S., Benchat N. (2010). Int. J. Electrochem. Sci..

[cit36] Kouakou V., Niamien P. M., Yapo A. J., Trokourey A. (2016). Chem. Sci. Rev. Lett..

[cit37] Singh A. K., Quraishi M. A. (2010). Mater. Chem. Phys..

[cit38] Al-Qudah M. A., Hamaideh R. S., Al-Momani I. F., Al-Bataineh N. (2020). J. Surf. Sci. Technol..

[cit39] Al-Qudah M. A., Al-Keifi H. G., Al-Momani I. F., Abu-Orabi S. T. (2020). Int. J. Corros. Scale Inhib..

[cit40] Al-Bataineh N., Al-Qudah M. A., Abu-Orabi S., Bataineh T., Hamaideh R. S., Al-Momani I. F., Hijazi A. K. (2022). Corros. Sci. Technol..

[cit41] Karthik G., Sundaravadivelu M. (2017). J. Adhes. Sci. Technol..

[cit42] Khadom A. A., Yaro A. S., Kadhum A. A. H. (2010). J. Chil. Chem. Soc..

[cit43] Dawod F. A., Akpomie G. K., Abuh M. A. (2012). Int. J. Sci. Eng. Res..

[cit44] El-Awady A. A., Abd-El-Nabey B. A., Aziz S. G. (1992). J. Electrochem. Soc..

[cit45] Fouda A. S., Abd El-Aal A., Kandil A. B. (2006). Desalination.

[cit46] Yan Y., Weihua L., Lankun C., Hou B. (2008). Electrochim. Acta.

[cit47] Liu Z., Wang J., Qin Z., Xia D. H., Behnamian Y., Hu W., Tribollet B. (2025). Corros. Sci..

[cit48] Hou M., Pan C., Wang M., Xia D. H., Qin Z., Hu W. (2024). Electrochim. Acta.

[cit49] Pan C. C., Xia D. H., Hou M. Y., Qin Z., Xu Y., Behnamian Y., Hu W. (2024). Corros. Sci..

[cit50] Ma H., Chen S., Niu L., Shang S., Li S., Zhao S., Quan Z. (2001). J. Electrochem. Soc..

[cit51] Wedian F., Al-Qudah M. A., Al-Mazaideh G. M. (2017). Int. J. Electrochem. Sci..

[cit52] Al-Qudah M. A., Algethami F. K., Fodeh O. A., Al-Momani I. F., Bataineh T. T., Alakhras A. I., Al-Mazaideh G. M. (2024). Corros. Eng. Sci..

