# Supplementary figures and images for: Acteoside: a novel green inhibitor for the corrosion of copper in 1.0 M HNO3 solution: experimental and theoretical investigation
Source: RSC Adv. 2025 Mar 27;15(12):9335–47. doi: 10.1039/d5ra01657f (PMC11947902; doi:10.1039/d5ra01657f)

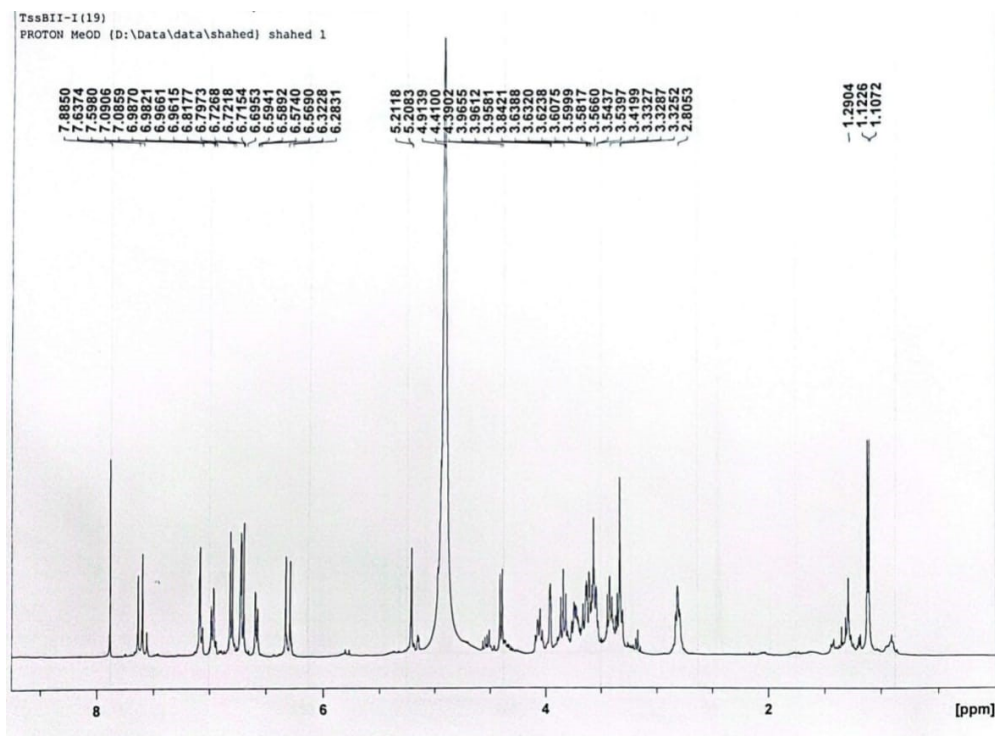

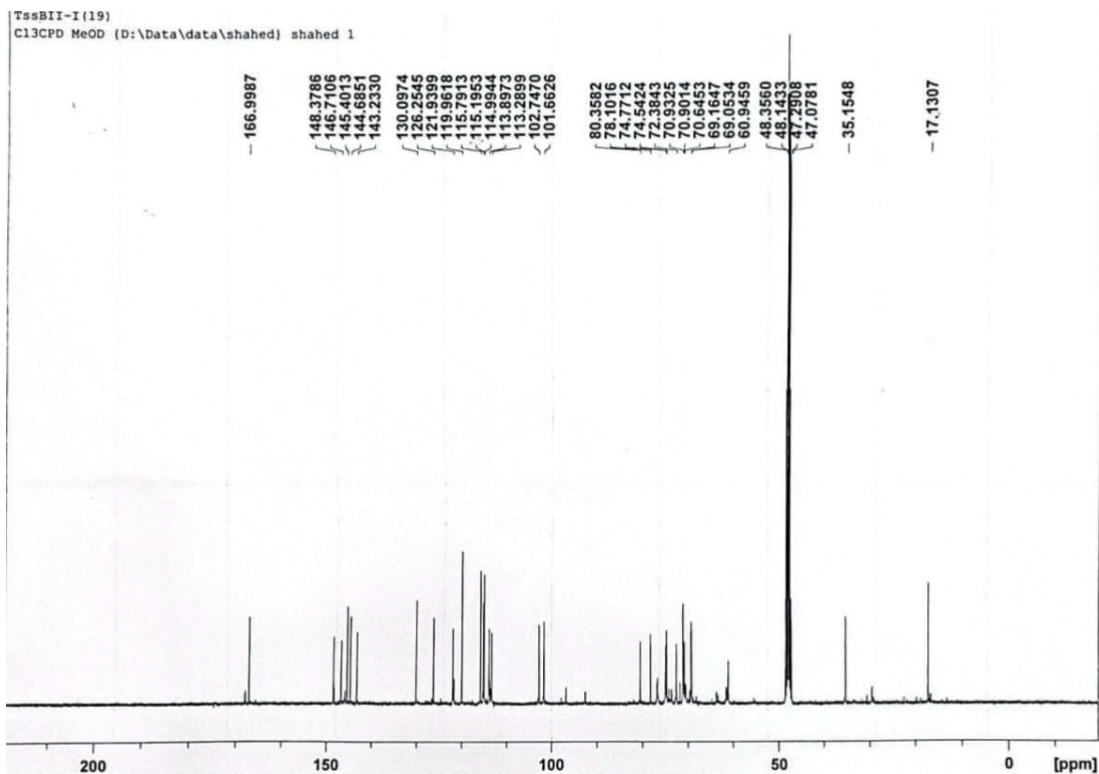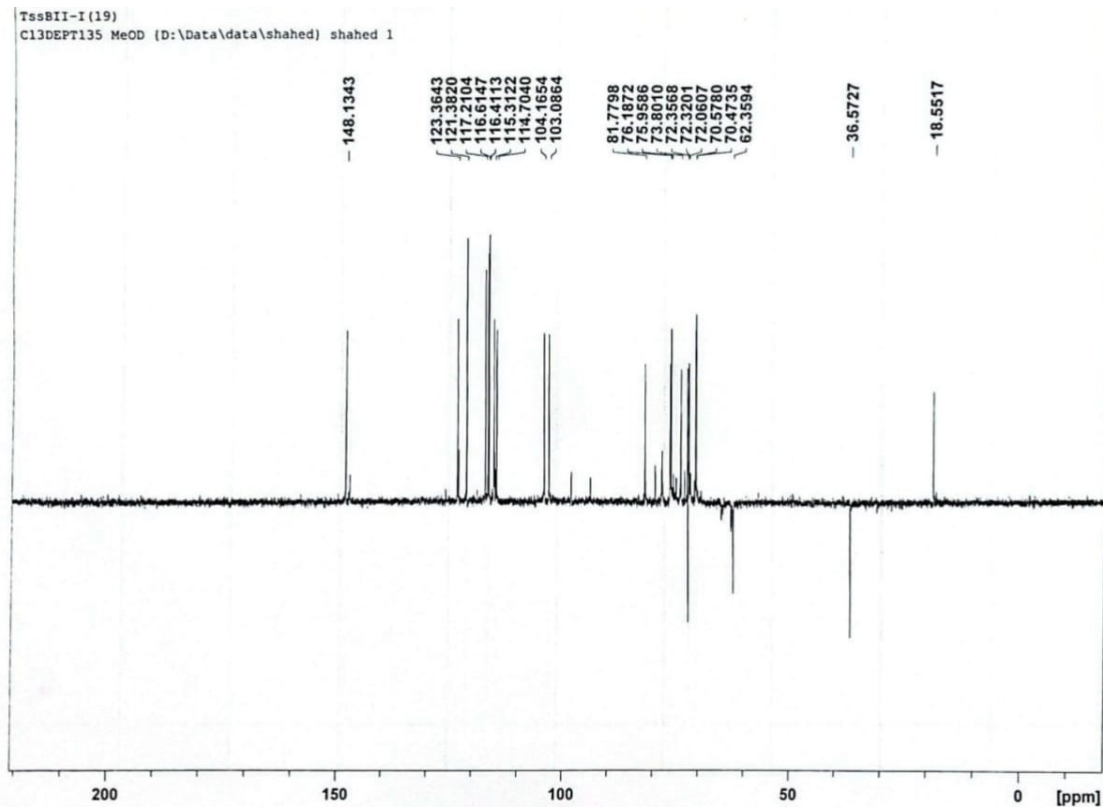

test  
C13DEPT90 MeOD (D:\Data\data\shahed) shahed 1

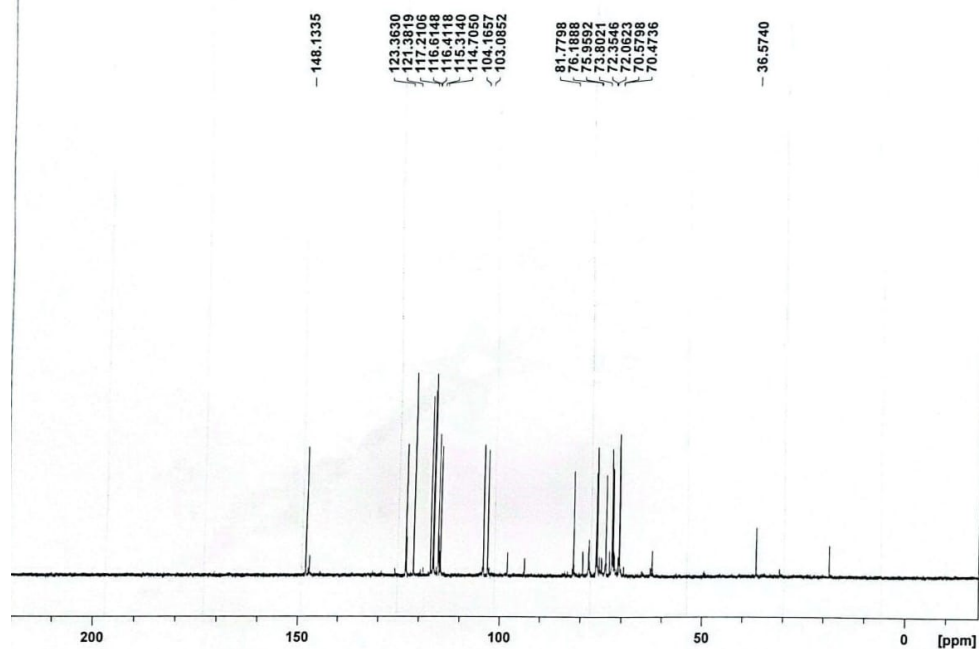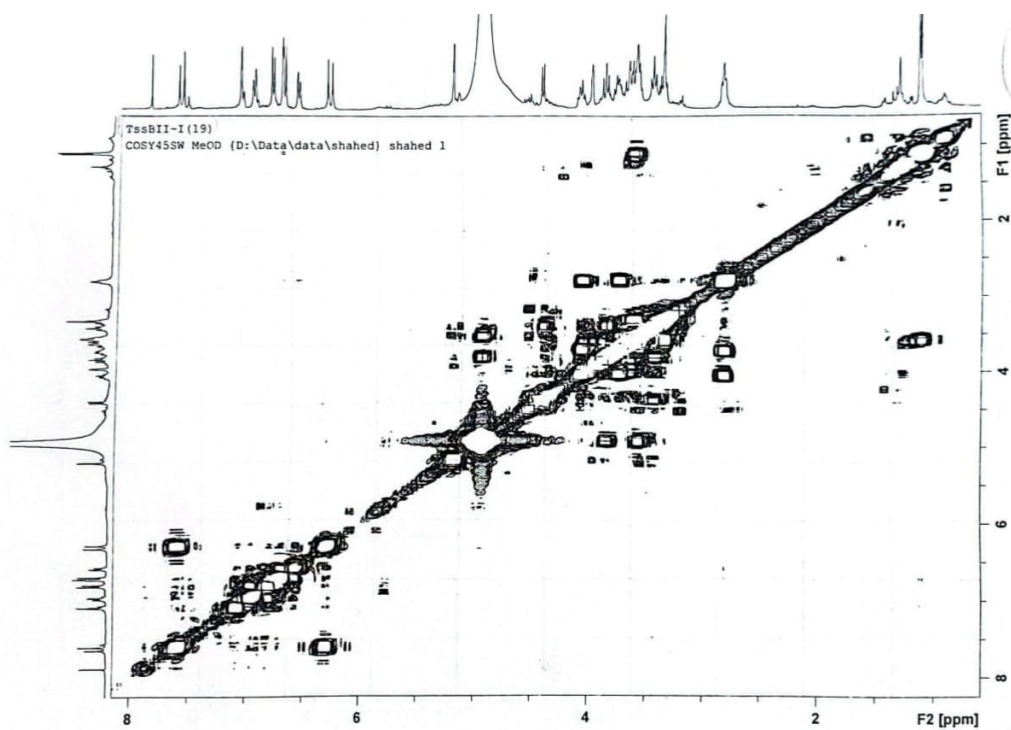

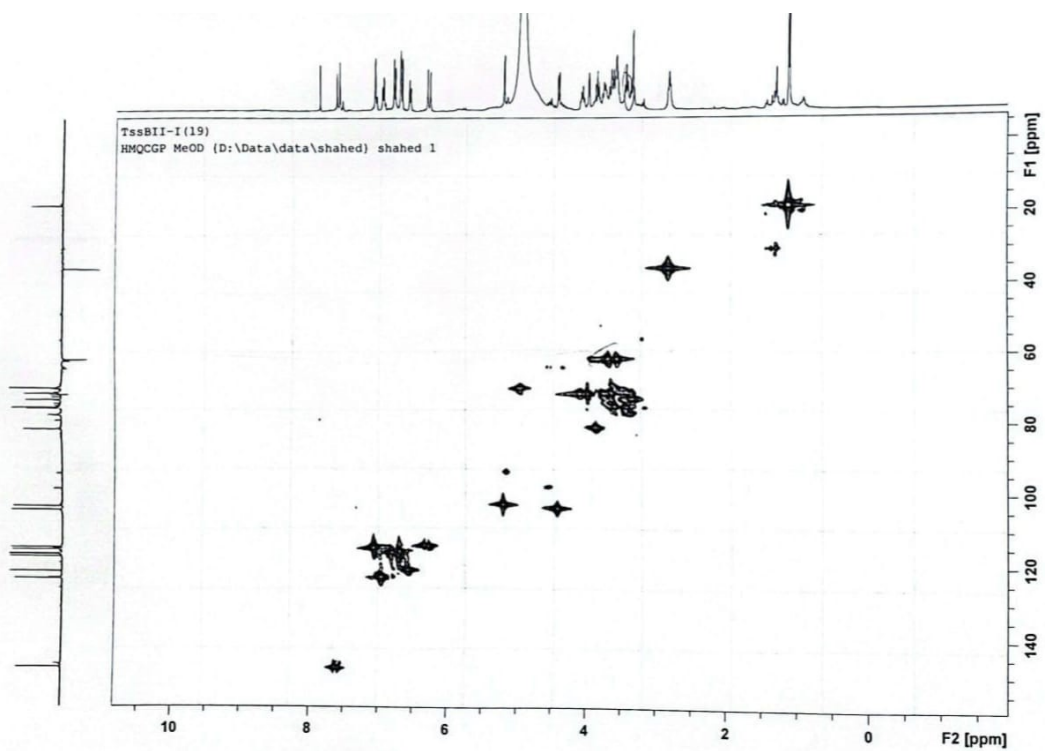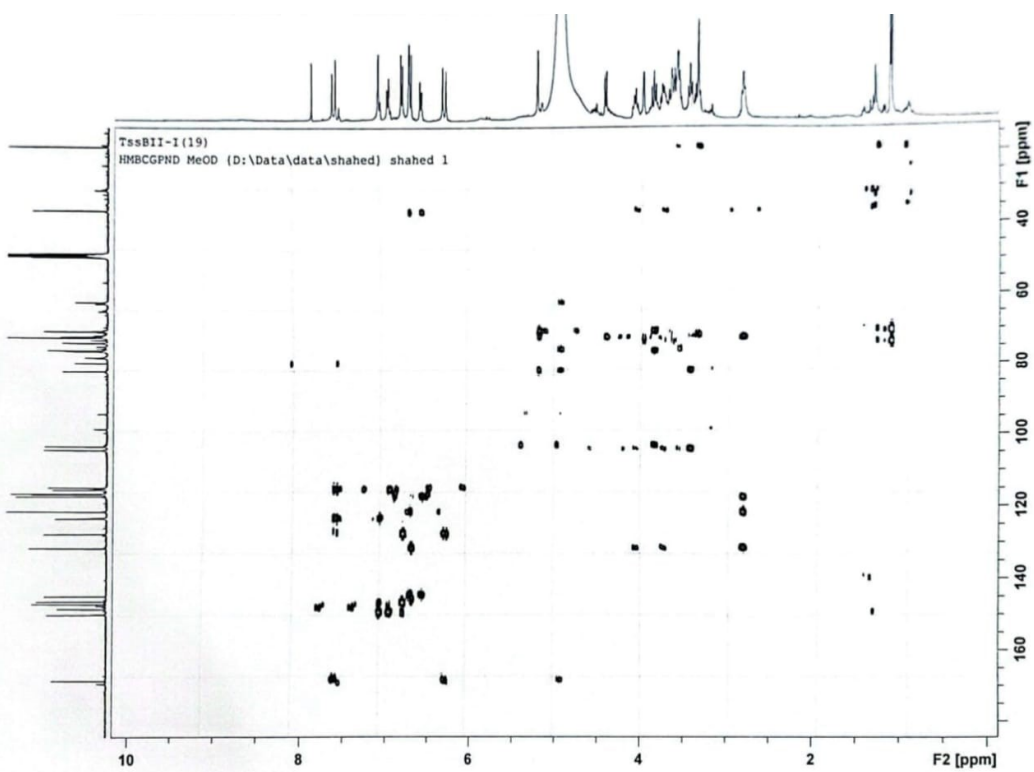

Supplement: RA-015-D5RA01657F-s001 [file RA-015-D5RA01657F-s001.pdf]
